#  Social inequalities in maternal mortality among the provinces of Ecuador

**DOI:** 10.26633/RPSP.2017.97

**Published:** 2017-05-17

**Authors:** Antonio Sanhueza, Jakeline Calle Roldán, Paulina Ríos-Quituizaca, Maria Cecilia Acuña, Isabel Espinosa

**Affiliations:** 1 Pan American Health Organization Pan American Health Organization Washington, D.C United States of America Pan American Health Organization, Washington, D.C., United States of America.; 2 Ministry of Public Health of Ecuador Ministry of Public Health of Ecuador Quito Ecuador Ministry of Public Health of Ecuador, Quito, Ecuador.; 3 Facultad de Ciencias Médicas Universidad Central del Ecuador Quito Ecuador Facultad de Ciencias Médicas, Universidad Central del Ecuador, Quito, Ecuador.

**Keywords:** Maternal mortality, health inequalities, social determinants of health, health equity, Ecuador, Latin America, Mortalidad materna, desigualdades en la salud, determinantes sociales de la salud, equidad en salud, Ecuador, América Latina

## Abstract

**Objective:**

*This study set out to describe the association between the maternal mortality ratio (MMR) estimates and a set of socioeconomic indicators and compute the MMR inequalities among the provinces of Ecuador*.

**Method:**

*A cross-sectional ecological study was conducted, using data for 2014 from the country’s 24 provinces. The MMR estimate was calculated for each province, as well as the association and its strength between MMR and specific socioeconomic indicators. For the indicators that were found to be significantly associated with MMR, inequality measurements were computed*.

**Results:**

*Despite a relatively low MMR for Ecuador overall, ratios differed substantially among the provinces. Five socioeconomic indicators proved to be statistically significantly associated with MMR: total fertility rate, the percentage of indigenous population, the percentage of households with children who do not attend school, gross domestic product, and the percentage of houses with electrical service. Of these five, only three had MMR inequalities that were significant: total fertility rate, gross domestic product, and the percentage of households with electricity*.

**Conclusions:**

This study supports research arguing that national averages can be misleading, as they often hide differences among subgroups at the local level. The findings also suggest that MMR is significantly associated with some socioeconomic indicators, including ones linked with significant health outcome inequalities. In order to reduce health inequities, it is crucial that countries look beyond national averages and identify the subgroups being left behind, explore the particular social determinants that generate these health inequalities, and examine the specific barriers and other factors affecting the subgroups most vulnerable to maternal health inequalities.

## RESUMEN

**Objetivo:**

El propósito de este estudio fue describir la asociación entre la razón de mortalidad materna y un conjunto de indicadores socioeconómicos, y calcular las desigualdades en la razón de mortalidad maternal entre las distintas provincias del Ecuador.

**Métodos:**

Se consideró un estudio ecológico transversal utilizando datos provenientes de las 24 provincias de Ecuador en el 2014, calculándose la razón de mortalidad materna para cada provincia, así como estudiando la asociación y su fuerza entre la razón de mortalidad materna y el conjunto de los indicadores socioeconómicos. Se obtuvieron las medidas de la desigualdades para aquellos indicadores socioeconómicos que mostraron una asociación estadísticamente significativa con la mortalidad materna.

**Resultados:**

A pesar de que la razón de mortalidad materna en Ecuador es relativamente baja a nivel mundial, las razones de la mortalidad materna difieren mucho entre las provincias. Hubo cinco indicadores socioeconómicos que resultaron estar asociados siginificativamente con la razón de mortalidad materna: la tasa total de fecundidad, el porcentaje de población indígena, el porcentaje de hogares con niños que no asisten a la escuela, el producto interno bruto y el porcentaje de hogares con servicio eléctrico. De estos cinco, solo tres mostraron desigualdades estadísticamente significativas en la mortalidad materna: la tasa total de fecundidad, el producto interno bruto y el porcentaje de hogares con electricidad.

**Conclusiones:**

Este estudio respalda las investigaciones que sostienen que los promedios nacionales pueden ser engañosos, pues a menudo ocultan diferencias entre subgrupos a nivel local. Los resultados también indican que la razón de mortalidad materna esta asociada significativamente con algunos indicadores socioeconómicos, incluyendo algunos que resultaron en desigualdades significativas en salud materna. Para reducir las inequidades en materia de salud, es crucial que los países adopten un enfoque que trascienda a los promedios nacionales y detecten los subgrupos que van quedando rezagados, analicen los determinantes sociales particulares que generan esas desigualdades en materia de salud y examinen los obstáculos específicos y otros factores que afectan a los subgrupos más vulnerables a las desigualdades en salud materna.

The year 2015 marked the end of the Millennium Development Goal (MDG) era. For Ecuador, that period had been one of economic growth (1), improvements in water quality and in sanitation, and an expansion of social services, including health and education for millions of people. In terms of maternal mortality, Ecuador was one of the countries of Latin America and the Caribbean that experienced the steepest reductions in the maternal mortality ratio. For the country as a whole, the ratio decreased from 185 maternal deaths per 100 000 live births in 1990 to 64 deaths per 100 000 live births in 2015, a reduction of 65.4% (2). Despite these impressive economic and social advances at the national level, insufficient progress was made at the subnational level, with thousands of people still living in poverty and thousands of women losing their lives due to preventable pregnancy-related causes (3).

Today, Ecuador and other countries face a new global health agenda that prioritizes universal health and equity, including through the Sustainable Development Goals (SDGs) and the new Global Strategy for Women’s, Children’s and Adolescents’ Health (4). In order for these nations to take on these ambitious targets, they have to start looking beyond national averages and identify the subgroups that are being left behind. It is also important that the countries start exploring the specific barriers and other conditions affecting these subgroups, and identify the mechanisms generating current health inequalities.

To help Ecuador prepare for these tasks, this study had two objectives: (1) to describe the association between maternal mortality and a set of socioeconomic indicators and, (2) based on those socioeconomic indicators, to compute the inequalities in maternal mortality among the provinces of Ecuador, using data for the year 2014.

## MATERIALS AND METHODS

For this research, a cross-sectional ecological study was conducted in 2014, using data from the 24 provinces of Ecuador.

The variables considered in this study were one heath indicator, nine socioeconomic indicators, and one demographic indicator.

The one heath indicator was the maternal mortality ratio (MMR).

The nine socioeconomic indicators were: (1) total fertility rate; (2) percentage of indigenous population; (3) percentage of households with children who do not attend school; (4) gross domestic product (GDP) per capita; (5) average household income; (6) percentage of poverty with unsatisfied basic needs; (7) percentage of households with inadequate services (e.g., no connection to a piped water supply or to the sewer system or a septic tank); (8) percentage of households with electricity; and (9) average number of persons per bedroom.

The one demographic indicator was live births.

The indicators used in this study were derived from three key information sources (5–7) on socioeconomic conditions in Ecuador and its provinces.

Some of the associations between the socioeconomic indicators and the MMR are intuitive, but others may not be. For instance, the average number of people per bedroom is an indicator of the quality of life and of living space. It especially reflects overcrowding and thus social deprivation. Therefore, this indicator was considered a proxy for poverty. On the other hand, the relationship between fertility and social factors helps to put the fertility rate into a larger, overall context, including identifying the most vulnerable sectors.

The analyses included the MMR estimate for each province, which was calculated by dividing the number of maternal deaths by the total number of live births reported in the year 2014, expressed as the number of maternal deaths per 100 000 live births. A 95% confidence interval (CI) for the MMR in each province was also computed.

First, an exploratory data analysis was carried out in order to ascertain the MMR and the selected socioeconomic indicators in Ecuador. Then, a study of MMR inequality was conducted for each of the selected socioeconomic indicators by taking two steps: (1) study the association and its strength between MMR and each of the socioeconomic indicators and (2) for the socioeconomic indicators that were found to be significantly associated with MMR, compute the MMR inequality measurements.

A weighted least squares regression model was used to study the association between MMR and the socioeconomic indicators. The weights were the number of live births in each province. The strength of the association was assessed with the Pearson correlation coefficient. The bootstrap method was used by considering 2 000 resamples in order to estimate the Pearson correlation and its bias-corrected 95% CI.

Simple and complex measures of inequality were computed to explore the magnitude of MMR inequality for each socioeconomic indicator. The simple measures included computing the absolute difference (AD) and the relative ratio (RR) (8). The complex measures included the modified slope index of inequality (MSII) proposed by Bacallao in 2007 (9) (which is an absolute measure of inequality) and the Poisson relative slope index of inequality (PRSII) (which is a relative measure proposed for this work that utilizes the Poisson regression model) (10). We computed 95% CIs for all those inequality measurements.

In order to compute the AD and the RR, quintiles of provinces for each of the socioeconomic indicators were created. For the purpose of this study, the first quintile (Group 1) represents the most disadvantaged provinces in terms of the quintile (Group 5) represents the most advantaged provinces for the indicator. The AD and the RR indicate the gap between the most disadvantaged and the most advantaged groups. The AD and the RR were computed by, respectively, subtracting and dividing the MMR in each of these two groups. Higher values of AD and of RR indicate greater inequality in maternal mortality.

The MSII and PRSII were computed through regression model fitting. In order to fit the models, the complete data set of the provinces was first ordered by socioeconomic indicator status, from the most disadvantaged to the most advantaged.

The MSII is based on the estimated slope parameter by fitting a linear regression model that considers the MMR as the dependent variable and the *Ridit* (the cumulative relative position of each province with respect to the socioeconomic indicator, which ranges between 0 and 1) as the independent variable. The weighted least squares method was used in this case.

The MSII was computed as MSII = b (*Ridit_Min_* - *Ridit_Max_*), where b is the estimated slope parameter computed by fitting the linear regression model. The *Ridit_Min_* and the *Ridit_Max_* are, respectively, the observed minimum and maximum *Ridit* values. The theoretical minimum and maximum *Ridit* values are 0 and 1, so that MSII =-b.

The 95% CI for the MSII was computed as [b_U_ (*Ridit_Min_* - *Ridit_Max_*), b_L_ (*Ridit_Min_* - *Ridit_Max_*)], where b_L_ and b_U_ are, respectively, the lower and upper limits computed from the 95% CI for the slope parameter. In order to compute the PRSII, the Poisson regression model was fitted by considering the number of maternal deaths as the response variable and the*Ridit* as the independent variable.

The formula for the PRSII was PRSII = exp{b (*Ridit_Min_* - *Ridit_Max_*)}, where exp is the exponential function and b is the estimated slope parameter computed by fitting the Poisson regression model. *Ridit_Min_* and *Ridit_Max_* are, respectively, the observed minimum and maximum *Ridit* values. The theoretical minimum and maximum *Ridit* values are 0 and 1, so that the PRSII = exp{-b}.

The formula for the 95% CI for the PRSII was [exp{b_U_ (*Ridit_Min_* - *Ridit_Max_*)}, exp{b_L_ (*Ridit_Min_* - *Ridit_Max_*)}], where b_L_ and b_U_ are, respectively, the lower and upper limits of the 95% CI for the slope parameter computed by fitting the Poisson regression model.

Data entries were made in Microsoft Excel software, and the statistical analyses were carried out using SAS version 9.2 software. Graphs were generated using Tableau version 9.3 software.

Ethical review was not required for this study since it does not contain any human research data (we utilized data that are publicly available online).

## RESULTS

**FIGURE 1.**
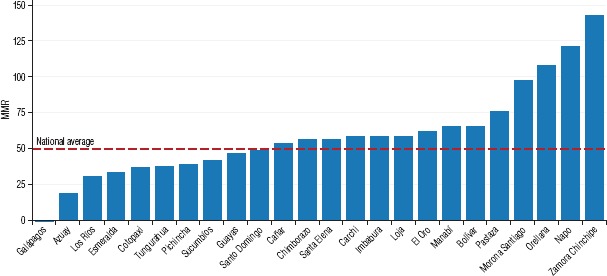
The maternal mortality ratio (MMR) (number of maternal deaths per 100 000 live births) in the provinces of Ecuador, in study of social inequalities in maternal mortality, 2014^a^

^a^ The maternal mortality ratio for Galápagos was 0 in 2014.

As of 2014, the estimated national MMR in Ecuador was 49.3 deaths per 100 000 live births. Despite this relatively low national average, 14 provinces had MMR estimates higher than that. The province with the lowest MMR was Azuay, with 19.1 deaths per 100 000 live births. The province with the highest MMR estimate was Zamora Chinchipe, with 142.2 deaths per 100 000 live births (Figure 1).

The average total fertility rate in Ecuador is 2.9 children per woman of childbearing age, ranging between 2.1 in Pichincha and 4.1 in Morona Santiago (Table 1).

Only 20% of the population overall is considered to be indigenous, with the percentage ranging from 0.1% in Manabí to 70% in Napo (Table 1). With respect to education, Ecuador is relatively well off. Overall, 0.9% of the households still have children who do not attend school; among the provinces, that ranges from 0.1% to 3.5% (Table 1).

The median GDP per capita in Ecuador is US$ 3 920.5, with values ranging from US$ 2 545 in Morona Santiago province to US$ 9 021 in Pichincha province. The median of the average household income per person is US$ 786.2, with Bolívar province having the lowest value (US$ 582.0) and Guayas province having the highest value (US$ 1 859.0). In terms of living in poverty with unsatisfied basic needs, that is true for 39.0% of the population overall, with values ranging from 16.6% in Pichincha province to 62.9% in Orellana province (Table 1).

In the case of housing conditions in Ecuador, the percentage of households with inadequate services ranges from 6.4% in El Oro province to 52.8% in Orellana province, with a national median of 22.2%. Further, although the overall percentage of households with electricity is relatively high in the country (94.0%), the percentage still ranges from 73.8% to 99.2% (Table 1).

**Figure 2:**
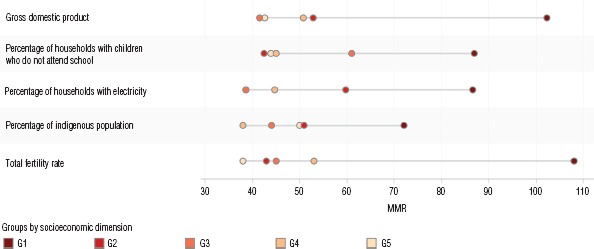
Disaggregated maternal mortality ratio (MMR) (number of maternal deaths per 100 000 live births) across the quintiles of socioeconomic indicators, in study of social inequalities in maternal mortality in Ecuador, 2014^a^

^a^ The first quintile, G1, represents the most disadvantaged provinces in terms of the socioeconomic indicator, and the fifth quintile, G5, represents the most advantaged provinces.

In terms of the association between the MMR and studied socioeconomic indicators in Ecuador, MMR was only statistically significantly associated with five socioeconomic indicators: total fertility rate, percentage of indigenous population, percentage of households with children who do not attend school, percentage of houses with electrical services, and GDP (Table 2). While three of them are positively associated (meaning the higher the value of the socioeconomic indicator, the higher the MMR), two of them are negatively associated (meaning the higher the value of the socioeconomic indicator, the lower the MMR). The socioeconomic indicators with the strongest associations with MMR are gross domestic product and total fertility rate. Figure 2 shows the distribution of MMR across the quintiles of provinces for each of these five socioeconomic indicators.

**TABLE 1.** Exploratory analysis of socioeconomic indicators in study of social inequalities in maternal mortality in Ecuador, 2014

Indicator	Minimum	Maximum	Mean	Standard deviation	Median
Gross domestic product (US$ per capita)	2 544.5	9 020.9	4 296.4	1 588.6	3 920.5
Household average monthly income (US$ per capita)	582.2	1 858.5	816.3	251.9	786.2
Poverty with unsatisfied basic needs (%)	16.6	62.9	39.0	14.1	37.8
Households with inadequate services (%)	6.4	52.8	25.6	15.5	22.2
Households with children who do not attend school(%)	0.1	3.5	1.0	0.7	0.9
Average number of persons per bedroom	1.5	2.2	1.8	0.2	1.8
Households with electricity (%)	73.8	99.2	94.0	0.1	96.3
Indigenous population (%)	0.1	70.0	20.0	20.0	10.0
Total fertility rate (births per women)	2.1	4.1	2.9	0.5	2.9

In terms of the association between MMR and total fertility rate, results indicate that they are positively associated (*P* value = 0.0031), which means that provinces with a higher total fertility rate also have a higher MMR. The strength of the association, which is provided by the correlation between these two variables, is 0.57 (95% CI [0.25, 0.79]), which is the second strongest association (Table 2).

Regarding the association between MMR and percentage of indigenous population, it is evident that they are positively associated, with a *P* value of 0.0256. The strength of the association is 0.38 (95% CI [0.06, 0.65]), which proves to be the weakest association out of the five socioeconomic indicators (Table 2).

Analyzing the association between MMR and the percentage of households with children who do not attend school, it is possible to conclude that they are positively associated (*P* value = 0.0189), with a 0.39 strength of association (95% CI [0.03, 0.65]) (Table 2).

Another socioeconomic indicator that proves to be statistically significantly associated with MMR in Ecuador is gross domestic product (*P* value = 0.001). Given that the strength of this association is -0.68 (95% CI [-0.84, -0.45]), one can conclude that the association is negative, which means that the provinces with a higher gross domestic product have a lower MMR. Out of the five socioeconomic indicators that are statistically significantly associated with MMR, gross domestic product has the strongest association (Table 2).

Another negative association can be found between MMR and the percentage of households with electricity (*P* value = 0.0039). The strength of the association is 0.44 (95% CI [-0.68, -0.10]) (Table 2).

Of the five socioeconomic indicators that were statistically associated with MMR, only three proved to have statistically significant MMR inequality measures when using the MSII and PRSII: total fertility rate, GDP, and the percentage of households with electricity (Table 3).

**TABLE 2.** Association between the maternal mortality ratio and socioeconomic indicators in study of social inequalities in maternal mortality in Ecuador, 2014

Indicator	Coefficient estimate	P value	Pearson correlation	Lower limit	Upper limit
Gross domestic product	-0.004	0.0138	-0.68	-0.84	-0.45
Average household income	-0.034	0.1205	0.24	-0.57	0.15
Poverty with unsatisfied basic needs	0.470	0.0633	0.34	-0.01	0.64
Percentage of households with inadequate services	0.462	0.0652	0.32	-0.02	0.63
Percentage of households with children who do not attend school	16.881	0.0189	0.39	0.03	0.65
Average persons per bedroom	21.698	0.3947	0.35	-0.03	0.64
Percentage of households with electricity	-232.380	0.0039	-0.44	-0.68	-0.10
Percentage of indigenous population	48.751	0.0256	0.38	0.06	0.65
Total fertility rate	23.842	0.0031	0.57	0.25	0.79

The AD between the group with the highest total fertility rate (Group 1) and that with the lowest total fertility rate (Group 5) was 70 (Table 4). This means that there are 70 additional maternal deaths per 100 000 live births occurring in the group of provinces with the highest total fertility rate than in the provinces with the lowest. Further, the RR of MMR between Group 1 and Group 5 was 2.8 (Table 4), indicating that women who live in the group of provinces that are the least advantaged in terms of this socioeconomic indicator are almost 3 times as likely to die from maternal causes as are women who are born in the group of provinces that are the most advantaged.

With respect to the total fertility rate for each province, the MSII value of 26.1 (Table 3) indicates that there are 26.1 more maternal deaths per 100 000 live births in the province with the highest total fertility rate (the most disadvantaged). The PRSII value of 1.7 (Table 3) indicates that women who live in the most disadvantaged province in terms of total fertility rate have almost twice the risk of dying from maternal causes as do women from the most advantaged province.

Regarding the MMR inequality that GDP produces, the AD value of 60 (Table 4) indicates that there are 60 additional maternal deaths per 100 000 live births occurring in the group with the lowest GDP (Group 1), compared to the group with the highest GDP (Group 5). The RR value of 2.4 (Table 4) indicates that women from Group 1 have more than twice the risk of dying from maternal causes as do women from Group 5.

Considering the values for each province in Ecuador, the MSII value of 28.8 (Table 3) indicates that there are approximately 32 additional maternal deaths per 100 000 live births occurring in the province with the lowest GDP than in the province with the highest GDP. Further, the PRSII value of 1.8 (Table 3) indicates that women from the most disadvantaged province are almost twice as likely to die from maternal causes as are women from the most advantaged province.

In terms of housing conditions, the AD value of 42 (Table 4) indicates that there are approximately 42 additional maternal deaths per 100 000 live births happening amongst women from the group with the lowest percentage of households with electricity (Group 1) than in the group with the highest percentage of households with electricity (Group 5). The RR value of 1.9 (Table 4) indicates that women from Group 1 have almost twice the risk of dying from maternal causes as do women from Group 5.

Considering all the values of each province, the MSII value of 22.6 (Table 3) indicates that there are an additional 23 women per 100 000 live births who die from maternal causes in the province considered most disadvantaged in terms of the percentage of households with electricity as compared to the women in the most advantaged province. Lastly, in terms of PRSII, the value of 1.6 (Table 3) indicates that women who are from the most disadvantaged province in terms of this socioeconomic indicator have a risk of dying from maternal causes that is 1.6 times as high as that of women from the most advantaged province.

## DISCUSSION

In recent years, Ecuador has made significant achievements in terms of overall economic growth, poverty reduction, and social service expansion. However, the country is still struggling to ensure that economic progress is made at all levels of society, and that improvements in effective social services coverage and health outcomes are shared by everyone, including the most vulnerable. Similarly, Ecuador also displays significant differences in terms of health outcomes, such as maternal mortality. For example, the province in Ecuador with the lowest MMR (Azuay, with 19.1 deaths per 100 000 live births) has an MMR that is similar to that of Trinidad and Tobago, which has one of the highest human development index (HDI) values in the Americas (8). In contrast, the province with the highest MMR (Zamora Chinchipe, with 142.2 deaths per 100 000 live births) has an MMR similar to that of the Solomon Islands (2), which is considered to have one of the lowest HDI values in Oceania (11).

**TABLE 3.** Complex measurements of inequality, with confidence intervals, for socioeconomic indicators, in study of social inequalities in maternal mortality in Ecuador, 2014

Socioeconomic indicator	Complex measurements (based on regression models)					
	Linear regression			Poisson regression		
	MSII^a^	Lower limit	Upper limit	PRSII^b^	Lower limit	Upper limit
Gross domestic product	28.8	7.7	50.0	1.8	1.1	2.9
Percentage of households with children who do not attend school	17.5	-7.1	42.2	1.4	0.8	2.4
Percentage of households with electricity	22.6	0.2	45.1	1.6	1.1	2.6
Percentage of indigenous population	5.0	-19.8	29.8	1.1	0.7	1.8
Total fertility rate	26.1	4.4	47.8	1.7	1.1	2.8

a MSII = modified slope index of inequality.

b PRSII = Poisson relative slope index of inequality.

**TABLE 4.** Simple measurements of inequality, with confidence intervals, for socioeconomic indicators, in study of social inequalities in maternal mortality in Ecuador, 2014

Socioeconomic indicator	Simple measurements (based on quintiles)					
	Absolute risk			Relative risk		
	AD^a^	Lower limit	Upper limit	RR^b^	Lower limit	Upper limit
Gross domestic product	60	14	106	2.4	1.5	4.0
Percentage of households with children who do not attend school	43	2	85	2.0	1.1	3.4
Percentage of households with electricity	42	-2	85	1.9	1.1	3.4
Percentage of indigenous population	22	-10	53	1.4	0.9	2.3
Total fertility rate	70	19	120	2.8	1.6	4.9

a AD = absolute difference.

b RR = relative risk.

The results presented in our study support previous research that argues that national averages often hide differences at the local level, and that these disparities can be strongly associated with different socioeconomic indicators (11). For example, while our statistical association study indicated that five of the nine socioeconomic indicators assessed were statistically significantly associated with MMR, the analysis using health inequality measures indicated that three of those five socioeconomic indicators (total fertility rate, GDP, and the percentage of households with electricity) were statistically significant in terms of the MMR inequalities.

In reviewing previous research, we have found that there are some studies on the association between socioeconomic indicators and maternal mortality in Latin American and Caribbean countries. However, few of these studies analyze the maternal mortality inequalities related to those socioeconomic factors. Our Ecuador study is important because it can provide decisionmakers with the type of information that is needed to determine which subgroups to target in order to reduce current health inequalities. The information can also provide insight into the specific mechanisms (social determinants) that generate these health inequalities. Furthermore, the evidence from this study can lead to additional research that examines the specific barriers and other factors affecting the subgroups most vulnerable to maternal health inequalities.

One of the strengths of this paper is the use of the Poisson regression model for computing a novel relative measure. On the other hand, there are some weaknesses in this study, such as the use of the cross-sectional ecological design that produces an ecological fallacy. This means that apparent associations between different provinces in Ecuador may not accurately reflect the true association between individuals within those provinces. However, inequality measurements using data for provinces (the first subnational level in Ecuador) provide relevant and more accurate information for the development of local health policies because they make it possible to identify the provinces that require equity-based interventions.

Limitations on the actual distribution of the maternal mortality data in the provinces by socioeconomics indicators may have also been a weakness in our analysis. As for the maternal mortality data, they come from the Epidemiological Surveillance System of the Ministry of Public Health (*Ministerio de Salud Pública* (MSP)). In dealing with a suspected case of maternal death, a multidisciplinary team carries out a thorough investigation to either confirm or rule out that initial analysis. Additionally, the vital records system (INEC in Spanish) and the MSP perform a semiannual process of active search for maternal deaths, through the review of death registers and local research processes.

The data on the socioeconomic indicators for 2014 were obtained from various sources, including the information system of the Central Bank of Ecuador and the INEC survey on urban employment unemployment, and underemployment.

One of the limitations of the statistical analysis is that maternal mortality data produced at the second subnational level (the cantons) are not taken into account. Thus, the results of the analysis in this work have to be considered as a first description of the maternal mortality inequalities, and further studies are needed. In a future study we will consider the use of multilevel analysis, by utilizing data from the cantons and provinces.

### Conclusions

This study is one of the few studies analyzing maternal mortality inequalities in Ecuador. By carrying out an analysis combining descriptive measures, association measures, and inequality measures, we hope to provide decisionmakers with the type of information needed for priority-setting. Further, the mixed analysis used in this article is innovative in the sense that it expands on the methodology traditionally used in studies that measure health inequalities, and it offers additional information that may enrich the understanding of maternal mortality inequalities within a country.

### Acknowledgments

We acknowledge the entire group working in the Gerencia de Mortalidad Materna at the Ministry of Public Health in Ecuador, and Dr. Gina Tambini and Dr. Adrian Diaz from the Pan American Health Organization (PAHO) Country Office in Ecuador. We would also like to thank Ramon Martinez, of the Department of Noncommunicable Diseases and Mental Health at PAHO, for his support of this work.

### Disclaimer

The authors hold sole responsibility for the views expressed, which may not necessarily reflect the opinion or policy of the *RPSP/PAJPH* or the Pan American Health Organization (PAHO).
